# METTL3 mediates m6A methylation modification of ULBP2 and affects the progression of cervical cancer

**DOI:** 10.1186/s41065-025-00483-8

**Published:** 2025-07-10

**Authors:** Hongtao Ren, Yuting Wang, Jiao Yu, Lei An, Xiulong Ma, Jiyuan Pan

**Affiliations:** 1 Department of Radiotherapy, Second Affiliated Hospital of Xi’an Jiaotong, University, Second Affiliated Hospital of Xi’an Jiaotong University Xinjiang Hospital, No. 157, Siwu Road, Xi’an, Shaanxi 710004 China; 2 Radiotherapy Center, People’s Hospital of Xinjiang Uygur Autonomous Region, Urumqi, Xinjiang 830001 China; 3 Radiotherapy Center, Shaanxi Provincical People’s Hospital, Xi’an, Shaanxi 710068 China

**Keywords:** ULBP2, METTL3, Cervical cancer, Radiotherapy, N6-methyladenosine

## Abstract

**Background:**

Cervical cancer (CC) is one of the most prevalent malignancies in women, posing a significant challenge globally. However, the precise molecular mechanism regulating CC progression through methyltransferase-like protein 3 (METTL3) and UL16 Binding Protein 2 (ULBP2) remains largely unknown.

**Methods:**

Bioinformatic analysis was used to identify the effect of ULBP2 expression in CC tissues. RT-qPCR and western blotting were employed to assess the mRNA and protein expression in CC cells and tissues. Methylthiazolyldiphenyl-tetrazolium bromide (MTT), 5‑Ethynyl‑2’‑deoxyuridine (EdU), wound healing, and transwell assays were utilized to estimate cell viability, proliferation, and metastasis, respectively. Cell apoptosis was detected by flow cytometry. CC cells were treated with different doses of radiotherapy. The m6A level was measured using methylated RNA immunoprecipitation (MeRIP) assay. A xenograft assay was conducted to further verify the roles of ULBP2 in CC.

**Results:**

ULBP2 was upregulated in CC. Downregulation of ULBP2 restrained the proliferation, metastasis and radiotherapy resistance of CC cells. METTL3 regulated m6A methylation modification of ULBP2. Insulin-like growth factor 2 mRNA binding protein 1 (IGF2BP1) promoted m6A methylation modification of ULBP2. METTL3 influenced the expression of ULBP2 and impacted the biological function of the CC cells. Silencing ULBP2 reduced the radioresistance of CC in vivo. Radiotherapy altered the gut microbiota in CC patients.

**Conclusion:**

METTL3 modulated the m6A methylation of ULBP2, affecting the oncogenic properties and radioresistance of CC cells.

**Supplementary Information:**

The online version contains supplementary material available at 10.1186/s41065-025-00483-8.

## Highlights

ULBP2 is highly expressed in cervical cancer.

Knockdown of ULBP2 inhibits proliferation, metastasis and radiotherapy resistance of cervical cancer cells.

METTL3 regulates m6A methylation modification of ULBP2.

METTL3 modulates the expression of ULBP2 and affects the physiological function of cervical cancer cells.

**Supplementary Information:**

The online version contains supplementary material available at 10.1186/s41065-025-00483-8.

## Introduction

Cervical cancer (CC) is one of the most common types of cancer among women worldwide, seriously affecting women’s health, especially in regions with limited resources where its prevalence is notably elevated [1, 2]. Radiation therapy serves as a fundamental treatment modality for CC management [3]. However, the primary reason for treatment failure in CC patients is often attributed to the radioresistance exhibited by cancer cells. Therefore, identifying novel targets associated with tumorigenesis and radioresistance is crucial for advancing CC treatment strategies.

UL16 Binding Protein 2 (ULBP2), a stress-inducible ligand encoded by human cytomegalovirus, functions as a critical activator of the NKG2D receptor on immune effector cells. This immunomodulatory molecule exhibits predominant expression in intestinal mucosal epithelia and neoplastic epithelial cells under pathophysiological stress conditions [4]. Several studies have demonstrated that the ULBP2 gene exhibits significantly elevated expression level in ovarian cancer and CC [5, 6]. Analysis revealed significantly increased ULBP2 protein level in CC specimens obtained from patients who had undergone radiation treatment [7]. Nevertheless, it is still undetermined whether ULBP2 boosts the radioresistance of CC.

N6-methyladenosine (m6A) stands as the predominant and extensively occurring post-transcriptional alteration in eukaryotic messenger RNA [8]. This modification is essential in regulating mRNA processing and is dynamically controlled through the reversible process involving demethylases (erasers), methyltransferases (writers), and proteins that recognize m6A (readers). METTL3, an important m6A writer, is a crucial methyltransferase and participates in several biological processes including proliferation and migration [9]. Insulin-like growth factor 2 mRNA binding proteins (IGF2BPs) stabilize mRNAs and promote translation by recognizing m6A modifications [10]. For instance, the inhibition of IGF2BP1 mitigates renal injury and inflammation by reducing m6A modifications and modulating the E2F1/MIF signaling pathway [11]. METTL3 plays a crucial role in enhancing cellular migration and invasive capabilities through the modulation of oncogene and tumor suppressor gene expression [12, 13]. Previous study has demonstrated that METTL3 exhibits elevated expression levels in CC and significantly contributes to its advancement [14]. However, whether METTL3 regulates the methylation modification of ULBP2 and influences the development of CC remains uncertain. Recent studies have demonstrated that the gut microbiota plays a critical role in tumor initiation and progression. Qin *et al*. [15] reported that phycocyanin mitigates radiation-induced pulmonary inflammation and fibrosis in mice by modulating the dysbiosis of lung and gut microbiota caused by radiotherapy. Notably, m6A modification has been shown to regulate host gene expression as well as signaling pathways associated with microbial metabolites. For instance, *Saccharomyces boulardii* has been shown to alleviate allergic asthma by restoring gut microbiota composition and metabolic balance through m6A-dependent upregulation of METTL3 [16]. *Fusobacterium nucleatum* has been reported to promote metastasis in colorectal cancer by downregulating METTL3 expression and reducing m6A methylation level [17]. Therefore, this study hypothesizes that the gut microbiota may indirectly regulate the levels of ULBP2 and influence the radioresistance of CC by modulating the expression of METTL3. Consequently, we also analyzed the dynamic changes in the gut microbiota of CC patients before and after radiotherapy to explore its potential role in the METTL3/ULBP2 axis.

Consequently, the research focused on exploring the critical functions and underlying mechanism of METTL3 and ULBP2 in the progression of CC, offering potential diagnostic markers and effective targets for CC treatment.

## Materials and methods

### Bioinformatic analysis

The expression level of ULBP2 mRNA in CC tissues and normal cervical tissues was collected from the cancer genome atlas (TCGA) database (https://tcga-data.nci.nih.gov) [18]. Using GEPIA (http://gepia.cancer-pku.cn/detail.php) [19], the expression differences of ULBP2 between cancerous and normal tissues were systematically analyzed. The prognostic significance of ULBP2 in CC was assessed using Kaplan-Meier analysis (http://kmplot.com/analysis/index.php?p=background) and ENCORI (http://starbase.sysu.edu.cn/index.php) [20]. The alpha diversity of gut microbiota of CC patients before radiotherapy (BR), during radiotherapy (DR) and after radiotherapy (AR) was evaluated using the Observed, Shannon, Simpson, and Pielou indices [21]. The beta diversity of the gut microbiota in CC patients was assessed at BR, DR, and AR using non-metric multidimensional scaling (NMDS) analysis [22]. A heatmap was created to visualize the proportional distribution of gut microbial communities in CC patients BR, DR, and AR.

### Sample collection

Tissue samples were collected from 32 patients undergoing surgical treatment for CC at Second Affiliated Hospital of Xi’an Jiaotong University. The collected samples comprised cancerous tissues and their corresponding adjacent non-cancerous tissues. None of the enrolled subjects had received prior anticancer treatments, including chemotherapy or radiation therapy, before undergoing surgical intervention. All collected tissue specimens were promptly preserved in liquid nitrogen for subsequent analysis. Written informed consent was acquired from all participants, and the research protocol received approval from the Ethics Committee of Second Affiliated Hospital of Xi’an Jiaotong University [No. XJTUAE2024-2215].

### Cell culture, irradiation and transfection

The immortalized cervical squamous epithelial cell model (Ect1/E6E7) and CC-derived cell lines (C-33 A, SiHa, HeLa) were acquired from the American Type Culture Collection (ATCC, Manassas, VA, USA). Cellular cultures were propagated in Dulbecco’s Modified Eagle Medium (DMEM, Gibco, Grand Island, NY, USA) containing 10% fetal bovine serum (FBS, Gibco) and antibiotic solution (100 U/mL penicillin-streptomycin, Gibco) under standard incubation conditions (37℃, 5% CO_2_). For irradiation, cells were seeded at 5 × 10^4^ cells/well in 6-well plates and exposed to X-ray radiation using a linear accelerator (Varian Medical Systems, USA) at a dose rate of 2 Gy/min, with total doses of 0 Gy, 2 Gy, 4 Gy, 6 Gy, or 8 Gy as indicated [23]. Short hairpin RNAs (shRNAs) targeting ULBP2, METTL3, METTL14, IGF2BP1 (sh-ULBP2, sh-METTL3, sh-METTL14, sh-IGF2BP1) and the respective negative control (sh-NC) were acquired from Keygen Biotech (Jiangsu, China). Sangon Biotech (Shanghai, China) provided the pcDNA3.1 vector carrying the ULBP2 gene (designated as OE-ULBP2) and the empty pcDNA3.1 vector (OE-NC). Transfection was performed using Lipofectamine 3000 reagent (Invitrogen, Carlsbad, CA), following the manufacturer’s guidelines [24].

### Real-time quantitative polymerase chain reaction (RT-qPCR)

Total RNA was extracted from cells and tissues using TRIzol reagent (Takara, Dalian, China). First-strand complementary DNA (cDNA) was synthesized using the PrimeScript RT reagent kit (Takara) following the manufacturer’s instructions. Quantitative real-time PCR was performed using SYBR Premix Ex Taq™ II (Takara) in a StepOnePlus™ Real-Time PCR system (Applied Biosystems). GAPDH served as the internal control for normalization. Relative gene expression levels were calculated using the 2^−ΔΔCT^ method [25]. The primer sequences were listed in Table 1.

**Table 1:** The primers used for RT-qPCR

Primer name	Primer sequence (5’-3’)
ULBP2 -F	TCCAGGCTCTCCTTCCATCA
ULBP2 -R	AGAAGGATCTTGGTAGCGGC
METTL3-F	CAGAGGCAGCATTGTCTCCA
METTL3-R	ATGGACACAGCATCAGTGGG
GAPDH-F	AGACGGGCGGAGAGAAACC
GAPDH-R	GAGAGAACAGTGAGCGCCTA

### Western blotting

Tissues and cells were lysed using RIPA buffer (Beyotime, Shanghai, China). Protein concentration was measured using a BCA protein assay kit (Beyotime). Equal amounts of total protein (30 µg) were separated by SDS-PAGE and transferred onto polyvinylidene difluoride membranes (Beyotime). The membranes were incubated overnight at 4 °C with the appropriate primary antibody, followed by incubation with an HRP-conjugated secondary antibody (1:1000, ab6702, Abcam, Cambridge, UK) for 1 h at room temperature [26]. The used primary antibodies (Abcam) were shown as below: ULBP2 (1:1000, ab275023), METTL3 (1:1000, ab240595), METTL14 (1:1000, ab309096), IGF2BP1 (1:1000, ab184305), GAPDH (1:1000, ab181602).

### Methylthiazolyldiphenyl-tetrazolium bromide (MTT) assay

Cell viability was detected by MTT assay. In brief, cells were plated at 1 × 10^3^ cells/well in 96-well plates and allowed to adhere overnight. After 24 h, MTT solution (5 mg/mL, Solarbio, Beijing, China) was added (20 µL/well), followed by 4 h incubation at 37 °C. After incubation, the medium was aspirated, and 150 µL of dimethyl sulfoxide (Solarbio) was introduced to each well. A microplate reader served to measure the absorbance at 570 nm [27].

### 5‑Ethynyl‑2’‑deoxyuridine (EdU) assay

Cell proliferation in CC cells was assessed using the EdU cell proliferation kit (RiboBio, Guangdong, China) [28]. Cells were seeded into 96-well plates at a density of 4 × 10^3^ cells per well and incubated overnight to allow for adherence. Subsequently, EdU reagent (50 µM) was added to the culture medium and incubated for 2 hours. Afterward, the cells were fixed in 4% paraformaldehyde (Solarbio) for 30 minutes, and permeabilized with 0.5% Triton X-100 (Solarbio) for 1 hour. The nuclei were labeled with 4’,6-diamidino-2-phenylindole (DAPI, Beyotime), and subsequently, the stained cells were visualized and captured using a confocal laser scanning microscope.

### Flow cytometry

Cells (1 × 10^6^) were resuspended in 500 µL of binding buffer and subjected to double staining with 10 µL of Annexin V-FITC and propidium iodide (Beyotime) for 15 min under light-protected conditions. Apoptotic cells were subsequently quantified using a flow cytometer [29].

### Transwell assay

Cell invasion was determined with transwell assay. Transwell chambers were placed in 24-well plates and coated with diluted Matrigel (8-µm pore size, Corning, Tewksbury, MA, USA). Cells (2 × 10^4^) were placed in the upper chamber with 100 µL of serum-free medium, while the lower chamber was filled with DMEM supplemented with 10% FBS (Gibco). After 24 h, cells on the top surface of the upper chamber were removed. The remaining cells were then fixed in 4% paraformaldehyde, followed by staining with 1% crystal violet (Beyotime). Cell staining was captured using an inverted microscope [30].

### Wound healing assay

Cell migration was evaluated using a wound healing assay. CC cells were plated in a 6-well plate with serum-free medium. Once they reached 95% confluence, a sterile 200-µL pipette tip was used to generate a wound by scratching the cell monolayer. Subsequently, the cell samples underwent three phosphate-buffered saline (PBS, Solarbio) washing cycles, followed by the addition of fresh medium for continued culture. Images were captured at 0 h and 24 h [31].

### Survival fraction analysis assay

Cells were seeded in 6-well plates at a density of 5 × 10^4^ cells per well and incubated for 24 h. After attachment, cells were irradiated with varying doses (0, 2, 4, 6, or 8 Gy) [23] using a linear accelerator (Varian Medical Systems, USA) at a dose rate of 2 Gy/min. The irradiation was performed 2 h after plating. Cells were incubated for 12 days to allow colony formation. Colonies were then fixed and stained with crystal violet, and the survival fraction (SF) was calculated as the ratio of colony counts in the irradiated group to the plating efficiency of unirradiated controls.

### Methylated RNA immunoprecipitation-qPCR (MeRIP-qPCR) analysis

Using the Magna MeRIP m6A Kit (RiboBio), MeRIP was conducted according to the manufacturer’s guidelines. The RNA was fragmented and immunoprecipitated with anti-m6A (1:1000, ab208577, Abcam) antibody or IgG (1:1000, ab172730, Abcam) using protein A/G magnetic beads. The bound RNA was reverse transcribed and analyzed by RT-qPCR [32].

### RNA immunoprecipitation-PCR (RIP-qPCR) assay

The RIP Kit (Geneseed, Guangzhou, China) was utilized according to the supplier’s protocol. Following transfection with sh-NC or sh-METTL3, cells were harvested and lysed using IP lysis buffer (Beyotime). Cell lysates were then incubated in RIP buffer along with magnetic beads conjugated with specific antibodies (anti-m6A, anti-IGF2BP1) or IgG. Protease and RNase inhibitors were applied to purify the lysates. Subsequently, RT-qPCR was performed to quantify the levels of the target RNAs [33].

### RNA stability assay

To assess RNA stability, actinomycin D (Act D, Sigma-Aldrich, STL, MO, USA) was administered to cells at a concentration of 5 µg/mL, followed by incubation at 37℃. RNA was extracted at the designated time points (0 h, 3 h, and 6 h) and quantified using RT-qPCR [34].

### Xenograft transplantation

BALB/c female nude mice (7 weeks old) were provided by ALF Biotech (Nanjing, China). The mice were randomly divided into four groups, with five mice in each group. For xenograft establishment, HeLa cells stably transfected with either sh-NC or sh-ULBP2 were harvested, and 5 × 10^5^ cells were subcutaneously injected into the right flank of each mouse. Tumor size was assessed every five days using vernier calipers. The volume of tumors was determined by applying the formula: volume = length×width^2^/2. The mice were subjected to X-ray irradiation as previously reported [23]. Mice were euthanized by CO_2_ asphyxiation, and tumor tissues were harvested for further analysis. Immunohistochemical staining was used to determine tumor proliferation. The standards of the Experimental Animal Ethics Committee of Second Affiliated Hospital of Xi’an Jiaotong University were adhered to all animal experiments [No. XJTUAE2024-2215].

### Statistical analysis

All data were analyzed using GraphPad Prism 8.0. Experimental data obtained from triplicate replicates were expressed as mean ± SD. The normality of data distribution was assessed using the Shapiro-Wilk test. Intergroup comparisons were statistically assessed using Student’s *t*-test for dual-group analyses or one-way ANOVA when evaluating multiple cohorts. A probability threshold of *P* < 0.05 was established for determining biological significance.

## Results

### ULBP2 is upregulated in CC

**Fig. 1:**
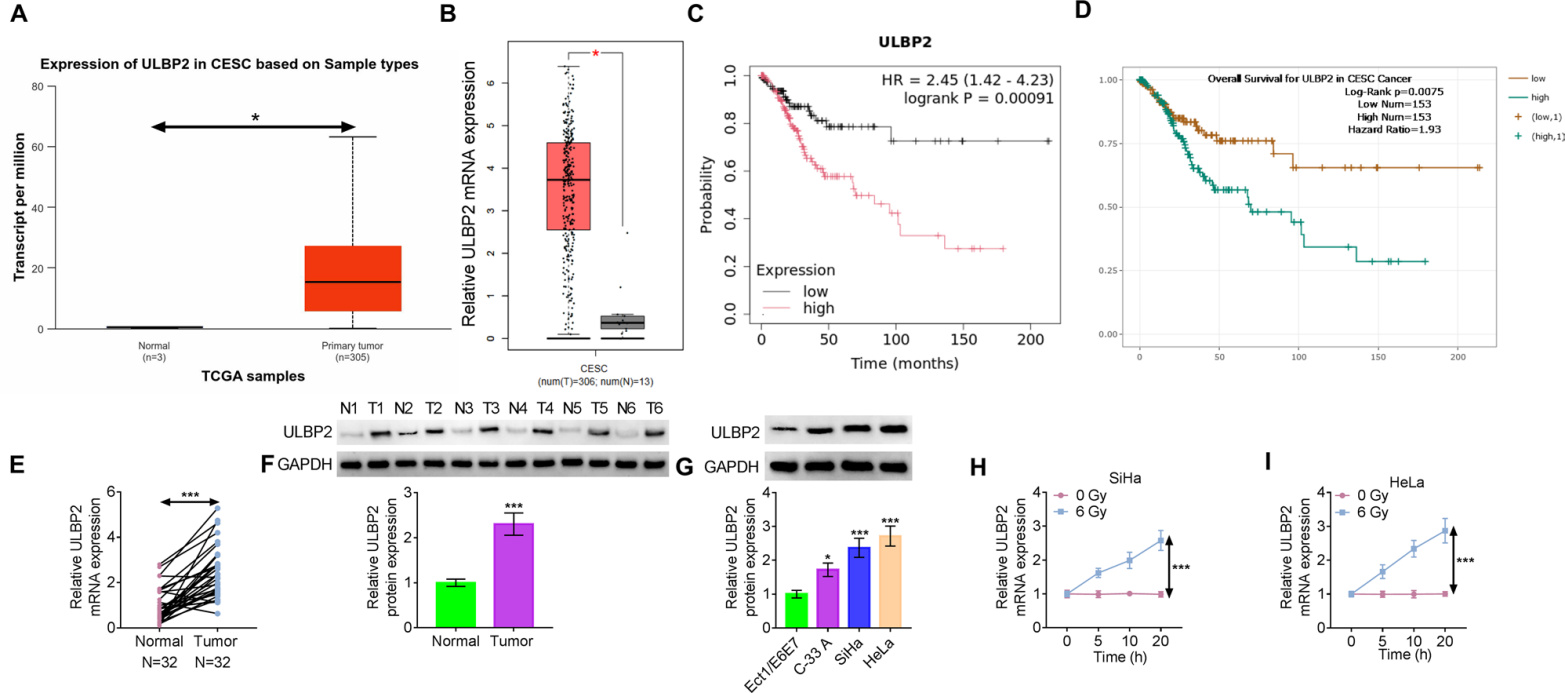
ULBP2 is upregulated in CC. (A) The expression of ULBP2 in CC was forecasted by the TCGA database. (B) The mRNA expression level of ULBP2 in the TCGA-GEPIA database. (C-D) ENCORI and Kaplan–Meier plotter websites showed the overall survival time of CC patients with high or low ULBP2 expression. (E) The mRNA expression level of ULBP2 in 32 CC and normal cervical tissues was detected by RT-qPCR. (F) The protein expression of ULBP2 in 6 CC and 6 normal cervical tissues was measured by western blotting. (G) Western blotting analysis was performed for ULBP2 level detection in CC cell lines and normal Ect1/E6E7 cells. (H-I) RT-qPCR analysis of ULBP2 expression in the SiHa and HeLa cells after treatment with a gradient of radiation doses. Data represent mean ± SD from three independent experiments. Statistical analysis: E-F was analyzed using an unpaired *t*-test, G-I was analyzed using one-way ANOVA. ^*^P < 0.05, ^***^P < 0.001

Initially, we accessed the TCGA database to analyze ULBP2 expression and observed higher expression level in CC tissues compared to normal tissues (Fig. 1A). The data obtained from the TCGA-GEPIA database also showed that ULBP2 was significantly overexpressed in CC (Fig. 1B). The ENCORI and Kaplan–Meier plotter websites indicated that high ULBP2 expression was associated with adverse prognosis of CC patients (Fig. 1C-D). The mRNA expression level of ULBP2 in CC tissues was greater than that in normal cervical tissues (Fig. 1E). We randomly selected 6 pairs of tissues and found that the expression of ULBP2 protein was increased in CC tissues (Fig. 1F). CC cell lines (C-33 A, SiHa, HeLa) exhibited a significant augmentation in ULBP2 protein level in contrast with normal Ect1/E6E7 cells (Fig. 1G). To investigate the impact of short-term radiation exposure on ULBP2, we examined ULBP2 expression after treating CC cells with a gradient of radiation doses. RT‐qPCR results confirmed that the expression of ULBP2 was substantially enhanced with an increasing radiation dose (Fig. 1H-I). SiHa and HeLa cells, which exhibited higher ULBP2 expression than C-33 A cells, were selected as study subjects.

### Silencing ULBP2 suppresses the proliferation, metastasis and radiotherapy resistance of CC cells

**Fig. 2:**
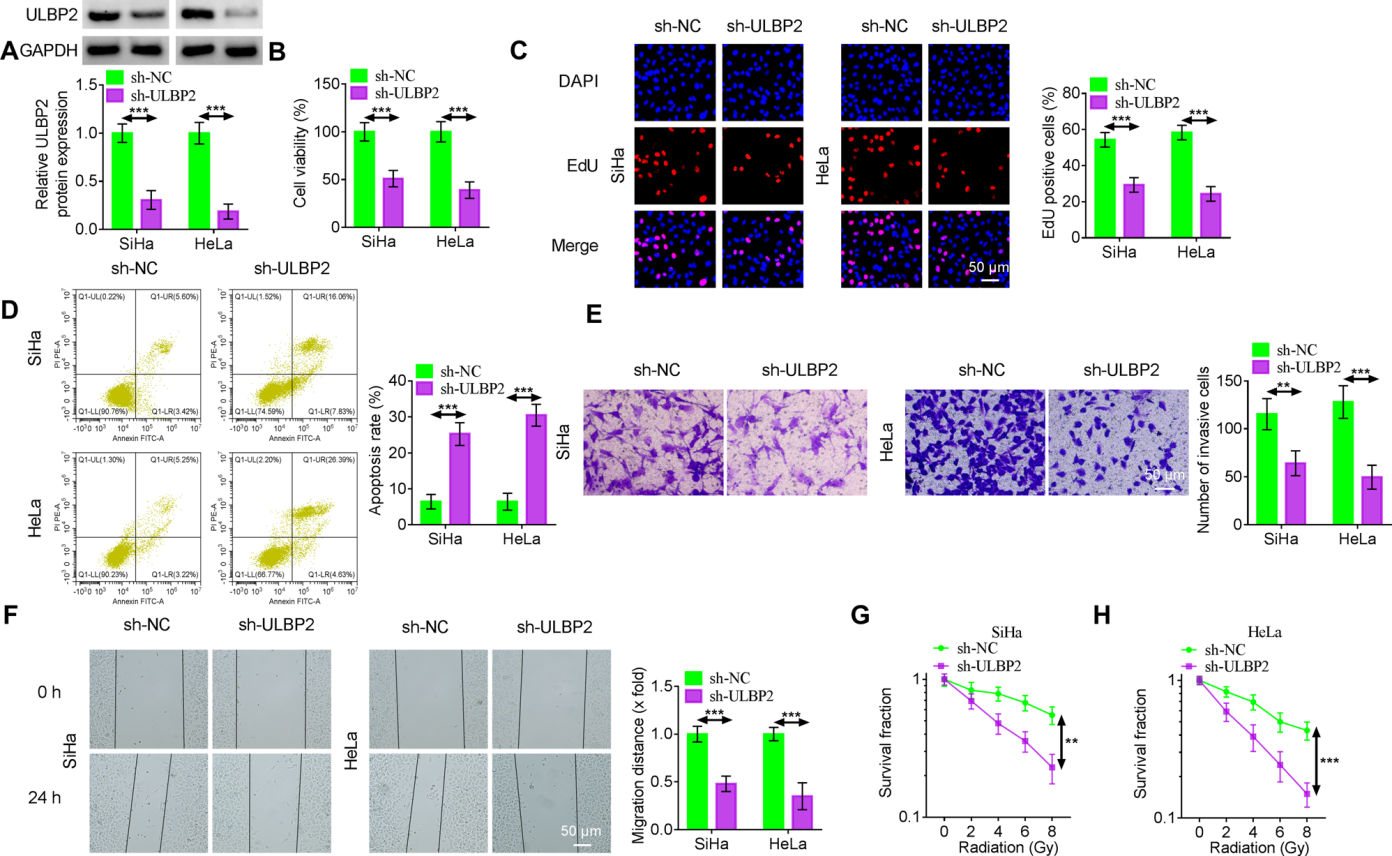
Silencing ULBP2 suppresses the proliferation, metastasis and radiotherapy resistance of CC cells. (A) The expression of ULBP2 in SiHa and HeLa cells transfected with sh-NC or sh-ULBP2 was detected by western blotting. (B) The effect of ULBP2 on SiHa and HeLa cell viability was measured by MTT assay. (C) Cell proliferation was detected by the EdU assay. Scale bar, 50 μm. (D) Flow cytometry was used to test the effect of ULBP2 on SiHa and HeLa cell apoptosis. (E) Transwell assay was utilized to examine the invasion of SiHa and HeLa cells. The cells were stained with crystal violet and then counted under a light microscope. Scale bar, 50 μm. (F) The result of wound healing assay for SiHa and HeLa cells knocking down ULBP2. Representative pictures were shown, and the width of the wounds was measured at 0 h and 24 h after the scratch. The migration distance was expressed as mean ± SD with SiHa and HeLa cells transfected with sh-NC as the standard. Scale bar, 50 μm. (G-H) Quantification of clonogenic assay was used to examine the effects of X-ray IR (0 Gy, 2 Gy, 4 Gy, 6 Gy, and 8 Gy) on cell growth of SiHa and HeLa cells with ULBP2 knockdown. Data represent mean ± SD from three independent experiments. Statistical analysis was performed using one-way ANOVA. ^**^P < 0.01, ^***^P < 0.001

In order to explore the function of ULBP2 in CC, we inhibited ULBP2 using sh-ULBP2 transfection in SiHa and HeLa cells. Western blotting confirmed that the transfection efficiency of sh-ULBP2 was satisfactory (Fig. 2A). The viability of SiHa and HeLa cells was decreased in the sh-ULBP2 group compared to the sh-NC group (Fig. 2B). In addition, the EdU assay demonstrated that knockdown of ULBP2 reduced cell proliferation (Fig. 2C). Silencing ULBP2 induced apoptosis in CC cells (Fig. 2D). Both the wound healing assay and transwell experiment results disclosed that downregulation of ULBP2 reduced cellular migration and invasion abilities (Fig. 2E-F). Knockdown of ULBP2 in SiHa and HeLa cells manifested a diminished survival fraction when the cells were exposed to X-ray IR (Fig. 2G-H).

### METTL3 mediates m6 A methylation modification of ULBP2

**Fig. 3:**
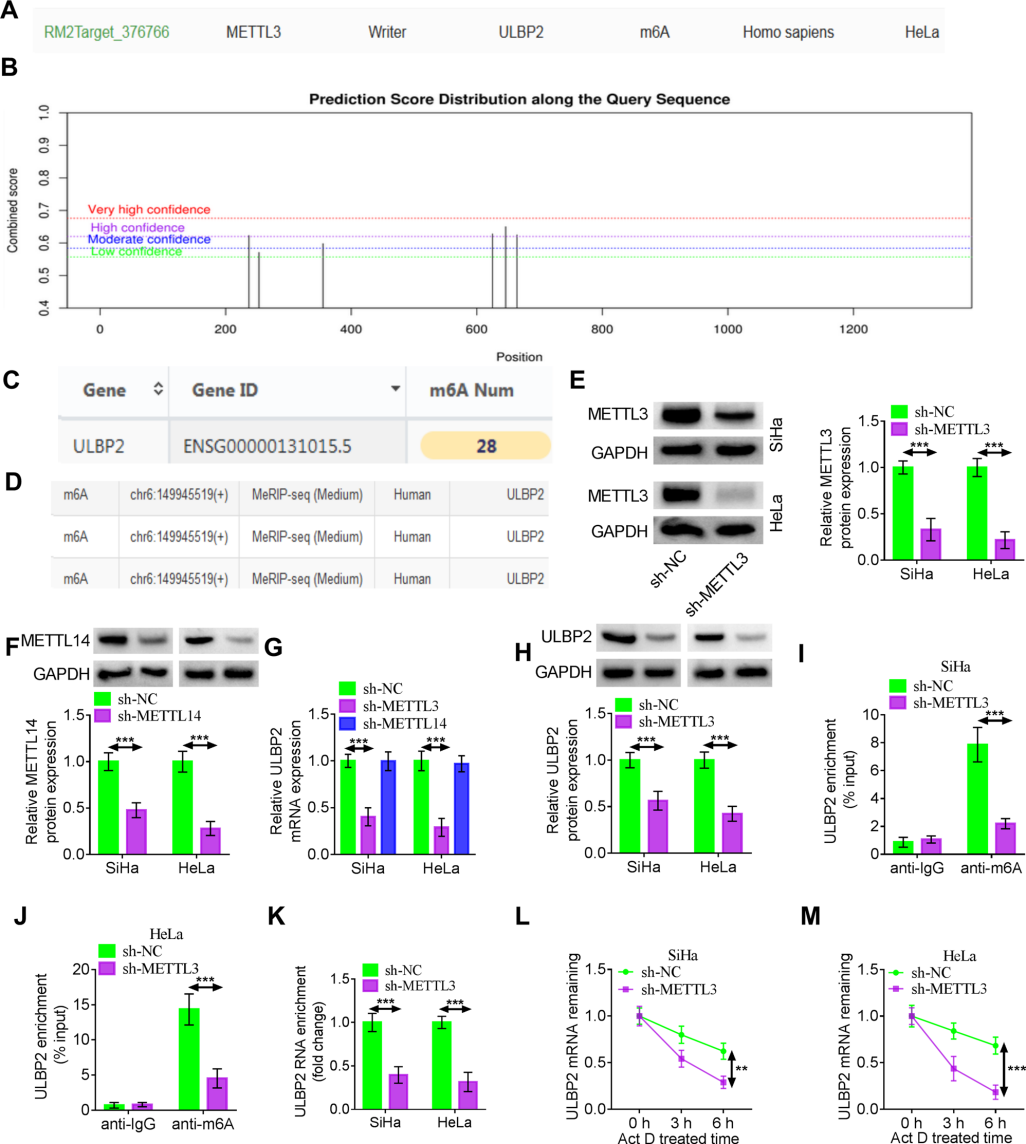
METTL3 mediates m6 A methylation modification of ULBP2. (A-D) The m6A locus of ULBP2 was predicted by the Rm2target (A), SRAMP (B), RMbase (C) and RMVar (D) databases. (E-F) Western blotting was employed to determine the protein expression of METTL3 or METTL14 after the transfection of sh-METTL3 or sh-METTL14 into SiHa and HeLa cells. (G) After knockdown of METTL3 or METTL14 in SiHa and HeLa cells, the mRNA level of ULBP2 was detected by RT-qPCR. (H) Western blotting was performed to assess ULBP2 protein expression in SiHa and HeLa cells transfected with either sh-NC or sh-METTL3 respectively. (I-J) MeRIP analysis of m6A modified ULBP2 mRNA in SiHa and HeLa cells with or without METTL3 knockdown. (K) RT-qPCR analysis of the RIP assay was used to detect the binding relationship between METTL3 and ULBP2. (L-M) The mRNA half-life of ULBP2 was detected in SiHa and HeLa cells after sh-METTL3 transfection and Act D treatment. Data represent mean ± SD from three independent experiments. Statistical analysis was performed using one-way ANOVA. ^**^P < 0.01, ^***^P < 0.001

To further investigate the mechanism of ULBP2 in CC, we used Rm2target (Fig. 3A), SRAMP (Fig. 3B), RMbase (Fig. 3C) and RMVar (Fig. 3D) databases to predict that ULBP2 contained m6A modification sites. As an essential component of the m6A methyltransferase complex, METTL14 cooperates with METTL3 and also significantly contributes to tumor progression in various cancer types [35, 36]. The protein expression of METTL3 or METTL14 in CC cells was decreased after transfection with sh-METTL3 or sh-METTL14 (Fig. 3E-F). Interference with METTL3 reduced the mRNA expression of ULBP2 in SiHa and HeLa cells, whereas METTL14 knockdown had no significant effect on ULBP2 mRNA level in these cells (Fig. 3G). Moreover, suppression of METTL3 restrained the protein expression of ULBP2 in CC cells (Fig. 3H). MeRIP-qPCR analysis showed the marked enrichment of ULBP2 m6A modification. Notably, METTL3 inhibition diminished ULBP2 m6A modification (Fig. 3I-J). RIP assay further revealed the binding relationship between METTL3 and ULBP2 mRNA (Fig. 3K). Next, we sought to determine whether METTL3-mediated m6A modification impacted ULBP2 mRNA stability. We treated SiHa and HeLa cells with the transcription inhibitor Act D and found that ULBP2 decayed faster in sh-METTL3-treated cells than in the sh-NC group (Fig. 3L-M).

### IGF2BP1 facilitates m6A methylation modification of ULBP2

**Fig. 4:**
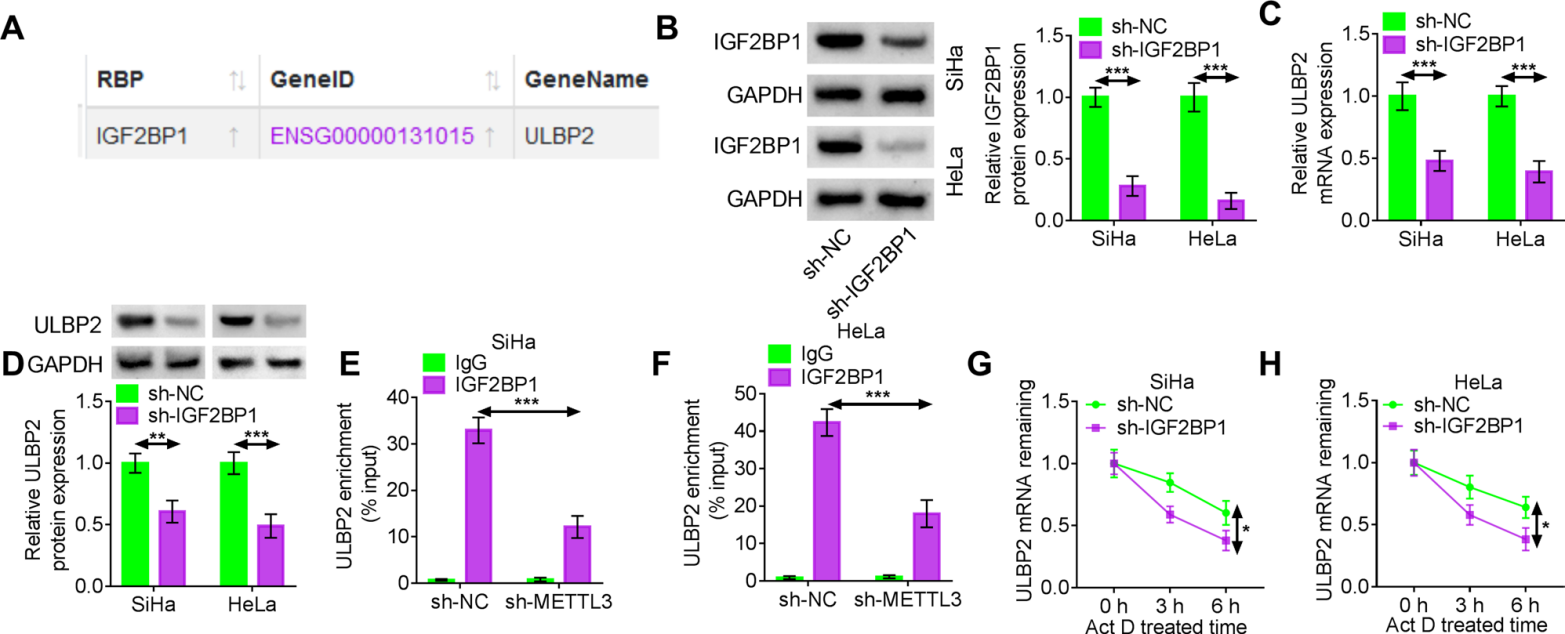
IGF2BP1 facilitates m6A methylation modification of ULBP2. (A) The interaction between IGF2BP1 and ULBP2 mRNA was predicted by ENCORI database. (B) Western blotting was applied to measure the protein expression of IGF2BP1 subsequent to the transfection of sh-NC or sh-IGF2BP1 into SiHa and HeLa cells. (C-D) The expression of ULBP2 mRNA and protein in SiHa and HeLa cells after the introduction of sh-NC and sh-IGF2BP1 was evaluated by RT-qPCR and western blotting. (E-F) RIP-qPCR analysis was adopted to verify the interaction between IGF2BP1 and ULBP2 after transfection of sh-NC or sh-METTL3 in SiHa and HeLa cells (G-H) The expression of ULBP2 in SiHa and HeLa cells transfected with sh-NC or sh-IGF2BP1 was detected by RT-qPCR at 0 h, 3 h and 6 h after ActD treatment. Data represent mean ± SD from three independent experiments. Statistical analysis was performed using one-way ANOVA. ^*^P < 0.05, ^**^P < 0.01, ^***^P < 0.001

Next, we identified an m6A reader that might be involved in regulating the stability of ULBP2 mRNA. The IGF2BP family regulates the stability of methylated mRNA by acting as an m6A reader [37]. We first used the ENCORI database to predict a potential interaction between IGF2BP1 and ULBP2 mRNA (Fig. 4A). To examine whether IGF2BP1 regulated ULBP2 expression, shRNA was employed to knock down IGF2BP1 in SiHa and HeLa cells. Transfection of sh-IGF2BP1 resulted in the obvious protein suppression of IGF2BP1 relative to sh-NC transfection (Fig. 4B). Silencing IGF2BP1 led to a marked decrease in both ULBP2 mRNA and protein expression (Fig. 4C-D). Subsequent RIP assay confirmed that the suppression of METTL3 notably diminished the binding affinity between IGF2BP1 and ULBP2 (Fig. 4E-F). Moreover, our findings illustrated that attenuation of IGF2BP1 promoted the degradation of remaining ULBP2 mRNA caused by Act D in both SiHa and HeLa cells (Fig. 4G-H).

### Overexpression of ULBP2 reverses the effects of Silencing METTL3 on the function of CC cells

**Fig. 5:**
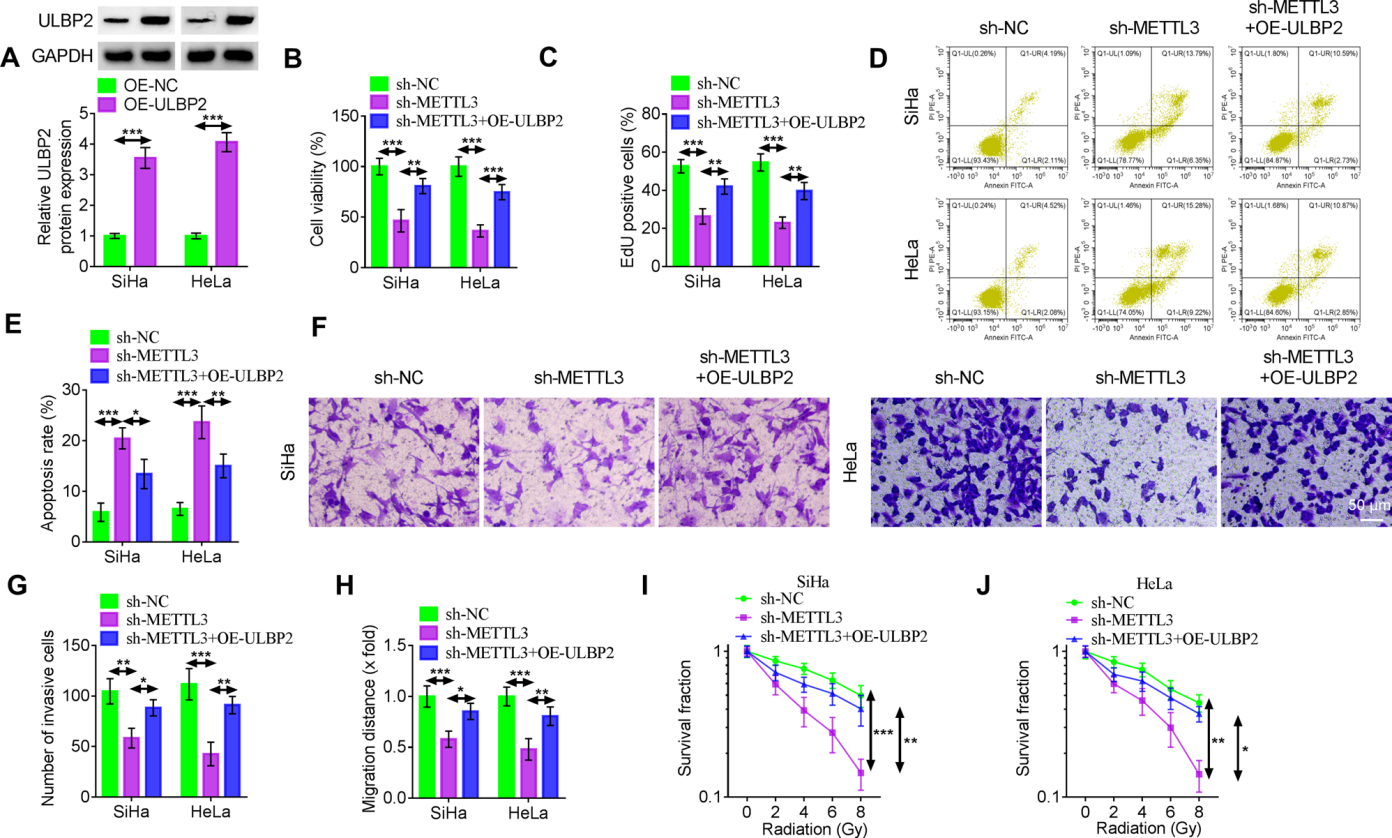
Overexpression of ULBP2 reverses the effects of silencing METTL3 on the function of CC cells. (A) Western blotting analysis was performed to evaluate ULBP2 protein expression in SiHa and HeLa cells following transfection with either OE-NC or OE-ULBP2. (B-J) SiHa and HeLa cells were transfected with sh-NC, sh-METTL3 or sh-METTL3 + OE-ULBP2. (B) The MTT assay was used for the detection of cell viability. (C) The EdU assay for cell proliferation was performed. (D-E) Apoptosis was assessed using flow cytometry. (F-G) Cell invasion was detected by transwell assay. Scale bar, 50 μm. (H) Wound healing assay was utilized to identify cell migration. (I-J) Colony formation assay for survival fraction in cells following exposure to 0 Gy, 2 Gy, 4 Gy, 6 Gy and 8 Gy irradiation. Data represent mean ± SD from three independent experiments. Statistical analysis was performed using one-way ANOVA. ^*^P < 0.05, ^**^P < 0.01, ^***^P < 0.001

To examine the role of METTL3/ULBP2 in CC, rescue experiments were conducted in SiHa and HeLa cells. Then, ULBP2-overexpressing vector was transfected into SiHa and HeLa cells. Compared with the OE-NC group, the expression of ULBP2 protein in the OE-ULBP2 group was increased (Fig. 5A). MTT and EdU assays showed that low METTL3 expression resulted in a significant decrease in cell proliferation. However, these effects were attenuated after reintroduction of ULBP2 (Fig. 5B-C). Additionally, apoptosis in sh-METTL3-transfected SiHa and HeLa cells was partially abrogated by transfection with OE-ULBP2 (Fig. 5D-E). Overexpression of ULBP2 recovered the inhibitory effect of knockdown of METTL3 on the invasion (Fig. 5F-G) and migration (Fig. 5H) of CC cells. Upregulation of ULBP2 mitigated the radiosensitivity of METTL3-deficient SiHa and HeLa cells (Fig. 5I-J).

### Knockdown of ULBP2 promotes the radiosensitivity of CC cells in vivo

**Fig. 6:**
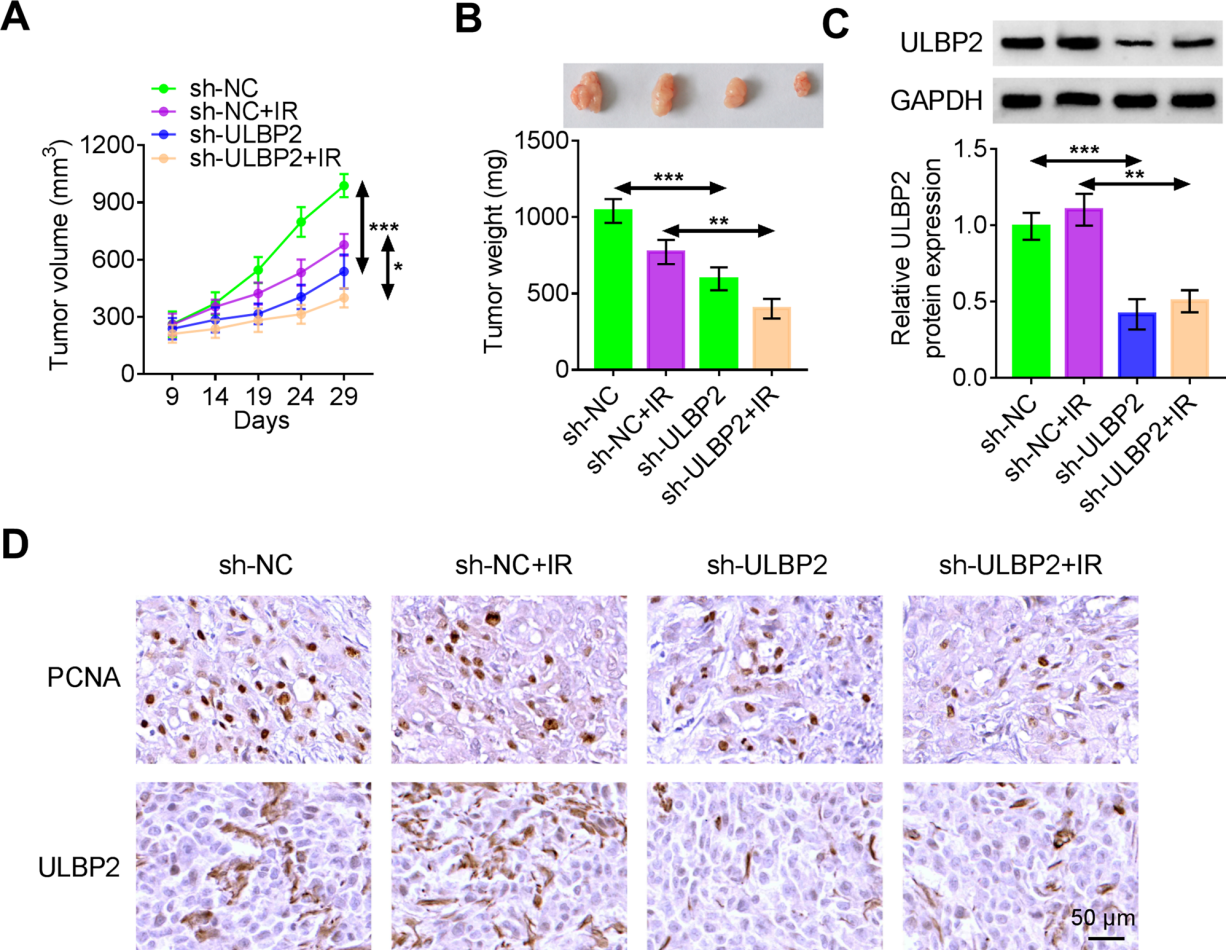
Knockdown of ULBP2 promotes the radiosensitivity of CC cells in vivo. (A-B) Photographs of excised subcutaneous tumors, tumor growth curves and tumor weight were shown for mice injected with HeLa cells stably transfected with sh-NC or sh-ULBP2, followed by exposure to irradiation or no treatment. (C) The protein expression of ULBP2 was detected by western blotting. (D) IHC images and protein expression of PCNA and ULBP2 in subcutaneous tumors resected in different groups. Scale bar, 50 μm. Data represent mean ± SD from three independent experiments. Statistical analysis was performed using one-way ANOVA. ^*^P < 0.05, ^**^P < 0.01, ^***^P < 0.001

To further confirm the radiosensitizing effect of ULBP2 depletion in CC cells under in vivo conditions, we constructed a xenograft model by subcutaneously inoculating HeLa cells into BALB/c nude mice. The mice were then irradiated and analyzed. Compared to the negative control group, tumor growth was inhibited in the ULBP2 knockdown group, particularly after irradiation (Fig. 6A-B). These results indicated that tumors in the ULBP2 depletion group showed enhanced sensitivity to irradiation. ULBP2 protein expression was downregulated in the sh-ULBP2 group relative to the sh-NC group. Similarly, the expression of ULBP2 protein in the sh-ULBP2 + IR group was lower than that in the sh-NC + IR group (Fig. 6C). IHC staining results revealed that the levels of PCNA and ULBP2 were decreased in the sh-ULBP2 group compared to the sh-NC group. Furthermore, the expression of both PCNA and ULBP2 was diminished in the sh-ULBP2 + IR group relative to the sh-NC + IR group (Fig. 6D).

### The gut microbiota is associated with the radiosensitivity of CC

**Fig. 7:**
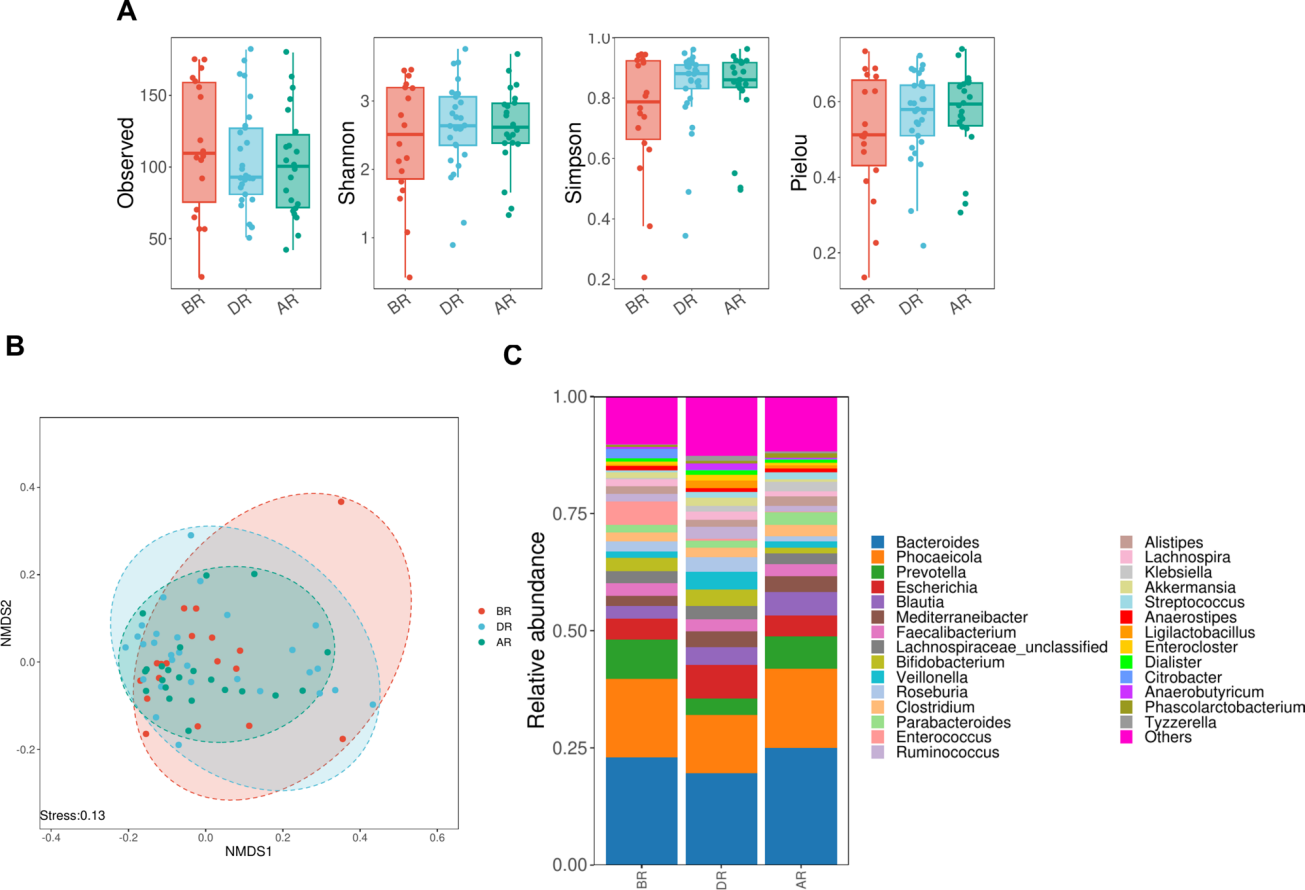
The gut microbiota is associated with the radiosensitivity of CC. (A) The dynamic changes of Observed index, Shannon index, Simpson index and Pielou index in DR and AR samples were compared with BR samples. (B) The NMDS plot of gut microbiota. Each data point represents the microbial community composition of one sample. (C) The relative abundance heat map of intestinal flora at the phylum level in BR, DR and AR samples

Scientists have detected that gut microbiota is intimately linked to the response to radiotherapy and proneness to toxic side effects [38]. To analyze the gut microbiota changes associated with radiotherapy, this study measured the Observed, Shannon, Simpson, and Pielou indices. These indices describe the richness, diversity, and evenness of microbial communities in CC patients BR, DR, and AR (Fig. 7A). In addition, we evaluated the similarity of microbial community structure by assessing the β diversity between samples. NMDS showed that the intestinal microbial spectrum between groups changed significantly (Fig. 7B). We assessed the bacterial abundance between groups at the phylum level and observed the marked differences of gut microbiota in each group of samples (Fig. 7C).

## Discussion

Radioresistance is the primary factor contributing to mortality in advanced CC [34]. This study revealed a previously uncharacterized regulatory mechanism by which METTL3 modulates the progression and radioresistance of CC through m6A methylation of ULBP2 mRNA. Our findings demonstrated that silencing ULBP2 enhanced cellular sensitivity to radiotherapy and suppressed tumor growth both in vitro and in vivo. Mechanistically, METTL3 promoted ULBP2 expression by enhancing its mRNA stability, an effect mediated through m6A-dependent recognition by the reader protein IGF2BP1.

ULBP2, a ligand for the natural killer group 2D receptor, has been previously implicated in tumor immune escape and prognosis across various cancers, including lung cancer, pancreatic cancer, and lymphoma [39, 40, 41]. By employing biological identification techniques, our findings revealed an elevated expression of ULBP2 in CC, which correlated with an adverse prognosis for CC patients. Radioresistance continues to pose a major challenge in improving the efficacy of radiotherapy for CC [42]. In our study, ULBP2 was upregulated in radioresistant CC cells, suggesting a potential link to radioresistance. Further functional experiments demonstrated that silencing ULBP2 attenuated malignant behaviors (proliferation, migration, invasion) in CC cells, concomitant with enhanced apoptotic activity. Additionally, the loss of ULBP2 increased the radiosensitivity of CC cells. Notably, in a nude mouse model, knocking down ULBP2 effectively suppressed radioresistance in vivo. These findings supported the hypothesis that ULBP2 functioned as an oncogenic factor in CC and contributed to therapeutic resistance.

M6A is widely recognized as a prevalent posttranscriptional modification in eukaryotic mRNAs and non-coding RNAs [43]. This modification influences the stability of related genes and contributes to tumorigenesis across various cancer types [44]. The m6A RNA modification is regulated by the m6A “writer” proteins (methyltransferases), “eraser” proteins (demethylases), and “reader” proteins. METTL14 plays an indispensable role in stabilizing the structure of METTL3 and enhancing its enzymatic activity by binding to RNA, which ultimately leads to an increase in m6A levels [45]. In the current work, we predicted the presence of m6A modification on ULBP2 through the website, and observed that the knockdown of METTL3 reduced ULBP2 expression, and knockdown of METTL14 did not affect the expression of ULBP2. Moreover, the expression of ULBP2 was demonstrated to be modulated by METTL3 through m6A-mediated mechanisms. Furthermore, we demonstrated that IGF2BP1 functions as the primary m6A reader responsible for stabilizing ULBP2 mRNA. IGF2BPs have been recognized as essential regulators of various biological processes, both under normal and stressed conditions [46]. For example, METTL3 was shown to inhibit senescence in human mesenchymal stem cells by stabilizing MIS12 mRNA via its interaction with the m6A-binding protein IGF2BP2 [47]. Based on our data, IGF2BP1 specifically interacted with ULBP2 transcripts, enhancing ULBP2 mRNA half-life through an m6A-mediated mechanism. In addition, rescue experiments manifested that upregulating ULBP2 alleviated the suppressive impact of METTL3 deletion on the aggressive phenotype of CC cells and their enhanced radiosensitivity.

In addition to uncovering the METTL3-ULBP2 regulatory axis, our study also explored the role of gut microbiota in CC progression under radiotherapy. A study involving 42 CC patients showed that microbial community profiling through 16 S rDNA sequencing identified distinct variations in α-diversity indices and β-diversity metrics between patients and healthy controls [48]. Through 16 S rDNA sequencing of fecal samples from patients before, during, and after radiotherapy, we observed significant alterations in microbial alpha and beta diversity. Notably, a shift in the abundance of certain bacterial genera was detected during treatment. Growing evidence suggests that microbial communities are closely associated with the physiological and pathological conditions of their host, including the initiation and progression of cancer, as well as the tumor’s sensitivity to treatment [49, 50]. Although the direct interaction between microbiota and the METTL3/ULBP2 axis requires further validation, our findings provide a rationale for considering gut microbiota as a potential modulator of therapeutic response in CC.

Taken together, our data supported a model in which METTL3-mediated m6A methylation enhanced the stability of ULBP2 mRNA via IGF2BP1, leading to elevated ULBP2 expression and increased CC cell malignancy and radioresistance. Moreover, radiotherapy-induced microbiota alterations might have indirectly contributed to these regulatory pathways, though further mechanistic studies were warranted. Clinically, targeting the METTL3-ULBP2 axis, either alone or in combination with microbiota modulation strategies, offered a promising avenue for improving CC treatment efficacy.

## Electronic supplementary material

Below is the link to the electronic supplementary material.

Supplementary Material 1

## Data Availability

No datasets were generated or analysed during the current study.

## Declarations

### Animal studies

Animal studies were performed in compliance with the ARRIVE guidelines and the Basel Declaration. All animals received humane care according to the National Institutes of Health (USA) guidelines. The standards of the Experimental Animal Ethics Committee of Second Affiliated Hospital of Xi’an Jiaotong University were adhered to all animal experiments [No. XJTUAE2024-2215].
