# Supplementary material for: METTL3 mediates m6A methylation modification of ULBP2 and affects the progression of cervical cancer
Source: Hereditas. 2025 Jul 10;162:123. doi: 10.1186/s41065-025-00483-8 (PMC12243326; doi:10.1186/s41065-025-00483-8)

The original western blots of Fig1

F

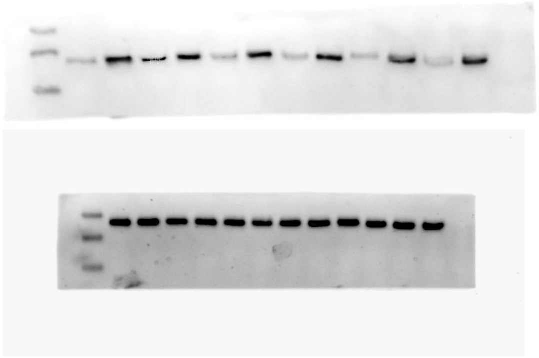

ULBP2

GAPDH

G

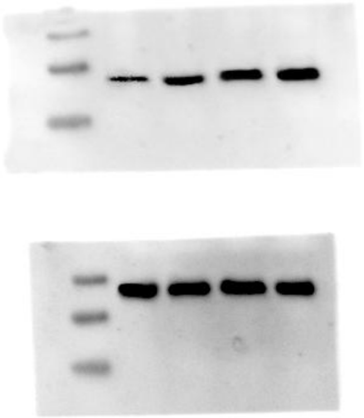

ULBP2

GAPDH

The original western blots of Fig2

A

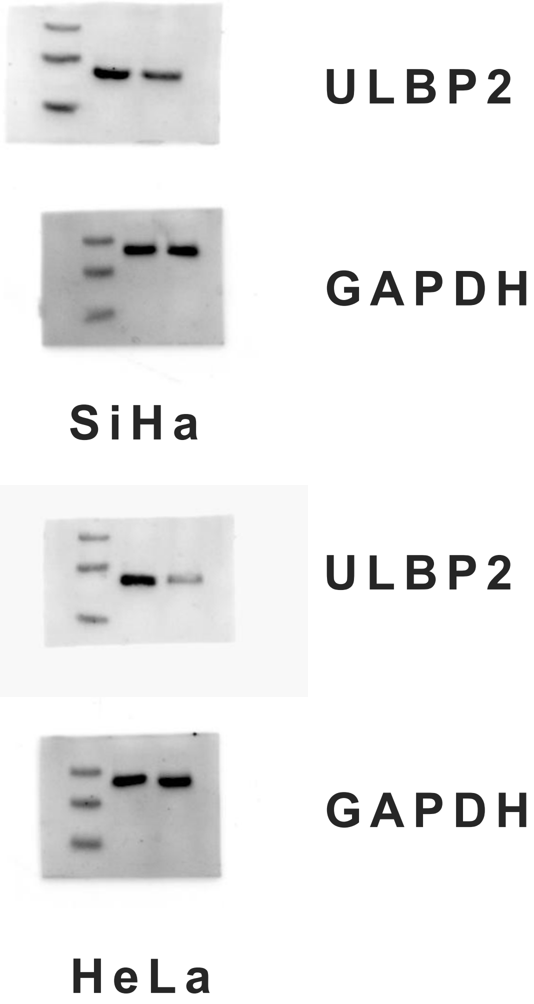

The original western blots of Fig3

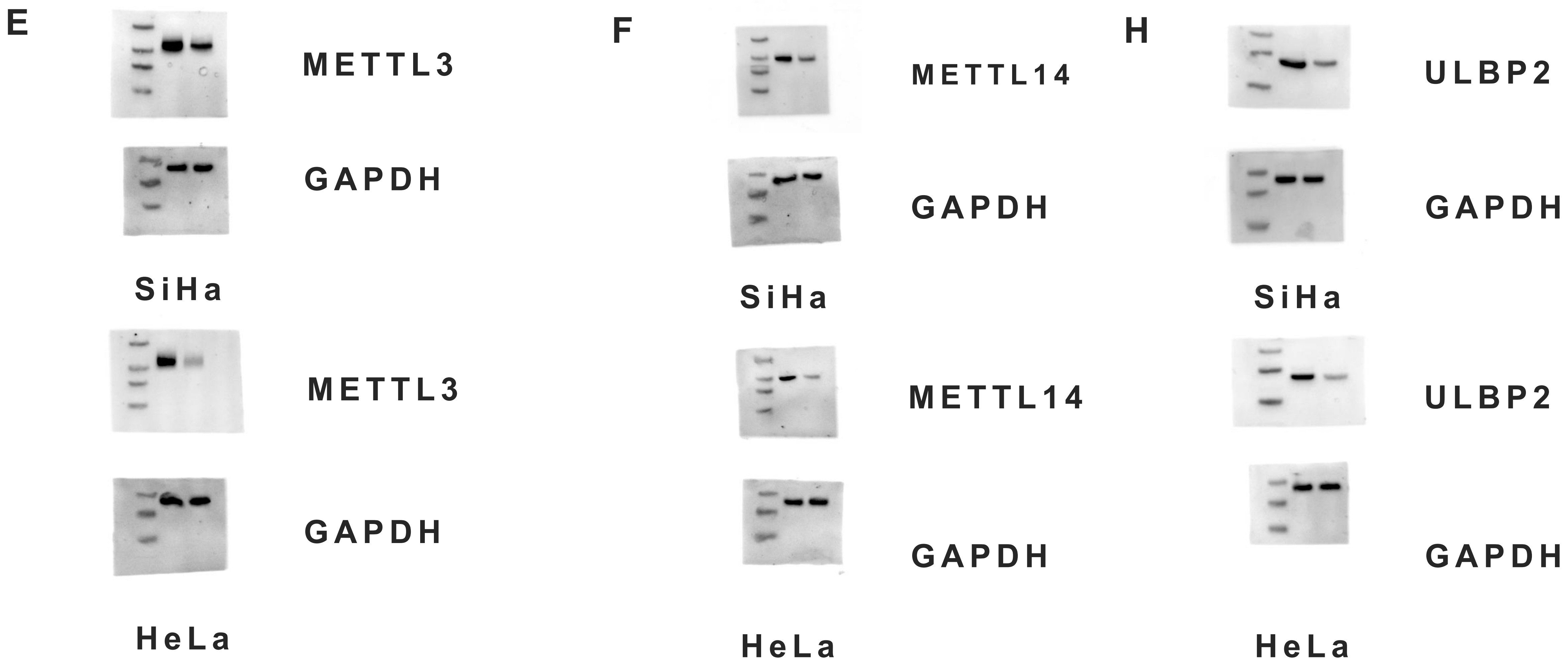

The original western blots of Fig4

B

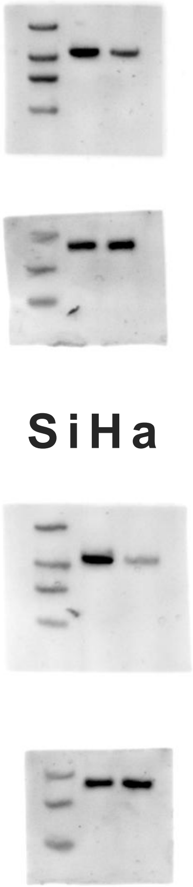

IGF2BP1

GAPDH

SiHa

IGF2BP1

GAPDH

HeLa

D

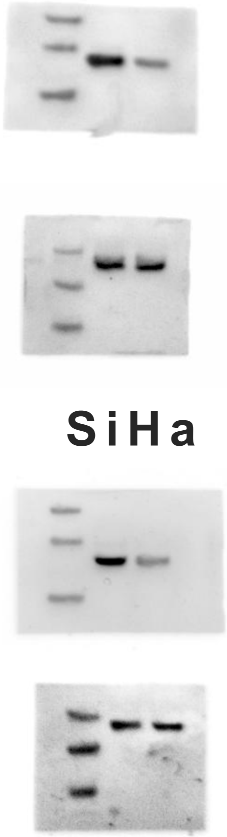

ULBP2

GAPDH

SiHa

ULBP2

GAPDH

HeLa

The original western blots of Fig5

A

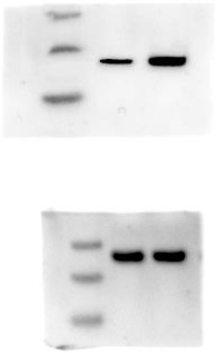

ULBP2

GAPDH

SiHa

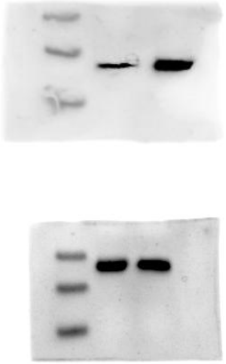

ULBP2

GAPDH

HeLa

The original western blots of Fig6

C

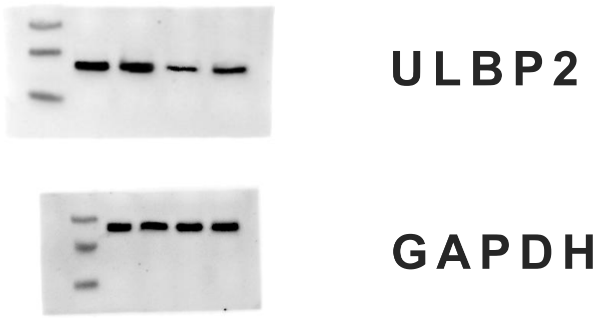

Supplement: Supplementary file 1 — Supplementary Material 1 [file 41065_2025_483_MOESM1_ESM.pdf]
